# Defining incidence and complications of fibrolamellar liver cancer through tiered computational analysis of clinical data

**DOI:** 10.1038/s41698-023-00371-2

**Published:** 2023-03-23

**Authors:** Travis Zack, Kurt P. Losert, Samantha M. Maisel, Jennifer Wild, Amin Yaqubie, Michael Herman, Jennifer J. Knox, Robert J. Mayer, Alan P. Venook, Atul Butte, Allison F. O’Neill, Ghassan K. Abou-Alfa, John D. Gordan

**Affiliations:** 1 Helen Diller Family Comprehensive Cancer Center (HDFCCC), University of California, San Francisco (UCSF), San Francisco, CA USA; 2 Bakar Computational Health Sciences Institute, University of California, San Francisco, CA USA; 3 Fibrolamellar Cancer Foundation, Greenwich, CT USA; 4 School of Medicine, University of California, Los Angeles, CA USA; 5 Memorial Sloan Kettering Cancer Center, New York, NY USA; 6 Department of Medical Oncology and Hematology, Princess Margaret Cancer Centre, University of Toronto, Toronto, ON Canada; 7 Dana-Farber Cancer Institute, Harvard Medical School, Boston, MA USA; 8 Dana-Farber Cancer Institute/Boston Children’s Cancer and Blood Disorders Center and Harvard Medical School, Department of Pediatric Oncology, Boston, MA USA; 9 Weill Medical College at Cornell University, New York, NY USA; 10 Quantitative Biosciences Institute, University of California, San Francisco, CA USA

**Keywords:** Cancer epidemiology, Liver cancer, Epidemiology

## Abstract

The incidence and biochemical consequences of rare tumor subtypes are often hard to study. Fibrolamellar liver cancer (FLC) is a rare malignancy affecting adolescents and young adults. To better characterize the incidence and biochemical consequences of this disease, we combined a comprehensive analysis of the electronic medical record and national payer data and found that FLC incidence is likely five to eight times higher than previous estimates. By employing unsupervised learning on clinical laboratory data from patients with hyperammonemia, we find that FLC-associated hyperammonemia mirrors metabolic dysregulation in urea cycle disorders. Our findings demonstrate that advanced computational analysis of rich clinical datasets can provide key clinical and biochemical insights into rare cancers.

Fibrolamellar liver cancer (FLC) is a rare primary liver cancer predominantly found in adolescents and young adults (AYA) without underlying liver disease^1^. Previously considered a morphological variant of hepatocellular carcinoma (HCC), FLC is now understood to be a separate entity with distinct molecular, histological, demographic, and clinical characteristics^2,3^. While systemic treatments approved for HCC are sometimes used for FLC, the significant molecular and mechanistic differences between the diseases suggest that each should have a distinct treatment paradigm^4^.

The Surveillance, Epidemiology, and End Results (SEER) Program is frequently relied upon for cancer incidence measurements, and reports an annual FLC incidence of 0.02 per 100,000 based on pathology reporting^5,6^. This rate is markedly lower than might be expected based on clinical practice observations, which suggest that FLC incidence may be approximately 1% of that of HCC^7–9^. Also, SEER data show a bimodal distribution in the age of FLC patients, with peaks at 15–19 years and 70–74 years, whereas disease experts find no other evidence for a major cluster of FLC patients above the AYA age range^4,10,11^. These older patients may instead have a recently defined variant of conventional HCC, marked by BAP1 mutation and lacking the FLC-associated *DNAJB1-PRKACA* fusion^12^. This suggests that underdiagnosis and misclassification both impact the registry data available for FLC through SEER and may hinder further attempts at mechanism-based treatment of this rare diagnosis.

Even though most FLC patients have normal underlying liver parenchyma, hyperammonemia is a frequent complication of the disease. Rather than liver insufficiency, hyperammonemia in FLC is thought to be a non-immunological paraneoplastic syndrome with features of urea cycle metabolism dysfunction^13–15^, though this mechanism remains unproven.

Applying a multimodal approach to comprehensive medical records from a large medical center (UCSF), combined with a large insurance claim dataset (Komodo Health) containing billing and diagnostic codes, we have a better elucidated national incidence of FLC and a potential unique biochemical mechanism for hyperammonemia in these patients:

To model the national incidence of FLC (Fig. 1A), we identified all patients with ICD-10-CM coding of “liver cell carcinoma” (LC, C22.0) or “other specified carcinoma of the liver” (C22.7) in the Komodo dataset. To increase the specificity for FLC within this cohort, we excluded patients with any of the 102 ICD codes associated with chronic liver inflammation (Table S1). In parallel, we confirmed 33 cases of FLC out of 4300 patients with ICD diagnoses of LC (0.6% of overall cases) within UCSF’s EMR between 2012 and 2021 through direct validation of clinical and pathology notes (Supplementary Information). Six of these patients had ICD codes excluded from our analysis, including diagnoses codes for hepatoblastoma, bile duct carcinoma, or sarcomas of the liver. Notably, the UCSF EMR includes 23 FLC patients from Northern California/Nevada in the last 10 years, representing an incidence of 0.017 per 100,000, nearly matching the SEER estimate. Given the existence of two other major academic centers in this area and other FLC patients who may never access tertiary care, this finding provides key proof of concept for our study. All UCSF FLC patients had an age at diagnosis below 50 years (mean 25.5 ± 8.3), whereas 87% of UCSF HCC patients were diagnosed at ages over 50 years (mean 61.96 ± 13.9). FLC accounted for 21% of LC cases in patients under 50 years old and 50% in patients under 30 years old without ICD-documented chronic liver inflammatory conditions.

**Fig. 1:**
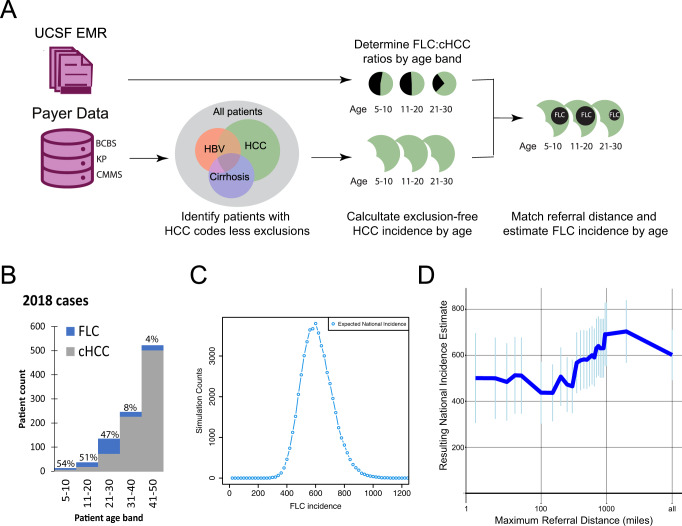
Bayesian inference of annual FLC incidence. **A** Analytical workflow: A subpopulation of patients with the “liver cancer” ICD-10 code but lacking other significant co-morbidities was created in the Komodo Healthcare Map, a national billing database. In parallel, the age-specific distribution of FLC vs. cHCC diagnoses was determined in the UCSF EMR using chart-validate FLC diagnoses. This distribution was then applied to the Komodo dataset to generate an overall annual incidence. **B** Relative proportion of FLC patients per age cohort on the 2018 incident population. **C** Empirically derived incidence distribution from our Monte Carlo simulation, illustrating confidence in the estimated annual cases. **D** FLC incidence estimations (±standard deviation) as in **C**, but excluding patients at different distances between the medical center and listed ZIP3 home address.

We used a Bayesian statistical method to map the relative rates of FLC and conventional HCC in the UCSF medical record onto the Komodo dataset to predict national incidence based on age and clinical covariates (Fig. 1B). After scaling, we calculated an annual FLC incidence of 602 ± 110 cases (Fig. 1C), about 0.185/100,000 individuals within the United States, nine times the previous estimate using SEER data. Since this estimate might be artificially inflated by referrals of FLC patients from further distances than our control population (HCC patients under 50 years old without co-diagnoses of liver disease; Supplementary Fig. S1), we repeated this calculation using used zip code data to create cohorts within different distances from our medical center (Fig. 1D). We found that, while the incidence estimate did vary based on referral distance, even the lowest estimate made was five times higher than current SEER data.

Case reports have described extreme hyperammonemia in FLC, but it is not always suspected in FLC patients with altered mental status. Thus, prospective analysis of ammonia levels was performed in two recent FLC clinical trials, showing 14.3–50% of tested patients with advanced FLC have elevated serum ammonia at baseline, with 27.3–71.3% having hyperammonemia at the end of treatment (Fig. 2A). Within the Komodo dataset, 372 or 8% of patients coded with LC without inflammatory liver disease also had a diagnosis code for high ammonia levels suggesting underdiagnosis (Fig. 2B).

**Fig. 2:**
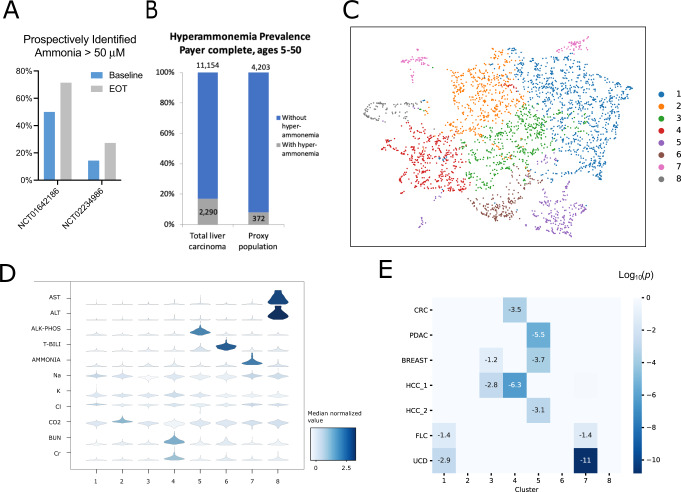
Characterizing FLC-associated hyperammonemia. **A** Incidence of hyperammonemia at baseline and end of treatment (EOT) for patients participating in two successive FLC clinical trials. Prospective assessment at the screening was instituted partially through NCT01642186 and performed from the initiation of NCT02234986. Patients were included in baseline and EOT cohorts if a value had been collected at that time. **B** Relative prevalence of hyperammonemia diagnostic coding in all liver carcinoma patients (e.g., including hepatitis diagnostic codes) vs. FLC proxy population, showing lower rates of detected hyperammonemia in the non-cirrhotic cohort. **C** Umap of entire concurrent complete metabolic profile results in all patients with laboratory evidence of hyperammonemia at UCSF, demonstrating subgroups of distinct clinical phenotypes. **D** Violin plots of individual lab values within each cluster from C; color shows median values while plot morphology indicates relative distribution. **E** Fisher exact test for enrichment of patients with selected diagnoses into clusters: Colorectal cancer (CRC), breast cancer, pancreatic cancer (PDAC), HCC with cirrhosis (HCC1), HCC without cirrhosis (HCC2), FLC, and primary urea cycle disorders (UCD).

Analysis of FLC patients with extreme hyperammonemia has suggested an association with urea cycle disorders (UCD), but it is not clear if this association extends to patients with intermediate levels of elevated ammonia. We hypothesized that patients would display distinct patterns of laboratory findings resulting from the metabolic basis of their hyperammonemia, enabling non-biased clustering based on their mechanism of disease. Of 3066 patient encounters with elevated ammonia levels and a complete metabolic panel (CMP) in the UCSF EMR, 19 were from FLC patients, 295 from cHCC patients, and 442 were from patients with primary UCD. We identified the natural partitions of lab values (Fig. 2C) using methods similar to those applied for cell type identification in single-cell RNAseq (see Supplementary Information). These clusters show distinct patterns of laboratory abnormalities. (Fig. 2D; Fig. S2; and Table S2).

We applied a Fisher exact test to assess the enrichment of specific diagnoses within each of these clusters. HCC patients were separated into those with co-diagnosis of cirrhosis (HCC1) and those without (HCC2). Interestingly, cirrhotic HCC patients were primarily enriched in the same cluster as patients with colorectal cancer (CRC), while non-cirrhotic HCC patients co-segregated with pancreatic (PDAC) and breast cancers. FLC patients did not cluster with other cancer patients and instead were co-enriched with primary UCD patients in cluster 1 (FDR-corrected 0.038, 0.001 respectively) and 7 (FDR-corrected *p* = 0.044, 1.4e^−11^; Fig. 2E). The combined enrichment FDR significance within these clusters was *p* = 0.0005 and *p* = 3.0e^−10^ for FLC and UCD encounters respectively. Cluster 7 was characterized by significantly higher ammonia levels than other clusters in the dataset. Cluster 1 was remarkable for relatively normal CMP, lacking reductions in sodium and chloride compared to other hyperammonemia clusters (Fig. S2).

Rare malignancies account for 20% of cancer incidence in the United States yet are poorly understood because of the challenges in assembling accurate patient cohorts and the frequent lack of specific ICD coding. Here we present a comprehensive analysis of large EMR and payer databases to define FLC incidence, suggesting that FLC is likely under-represented in SEER and underdiagnosed in young patients with liver cancer. Importantly, our Bayesian analysis was performed with highly stringent ICD-10 code exclusions, which removed 18% of FLC patients from the UCSF cohort, potentially lowering the estimated national incidence. Further, while our strategy risks overestimating due to referral bias of rare diseases to tertiary centers, we have refined our estimate based on patient distance from the tertiary center. This strategy of combining EMR and payer databases with adjustment for referral patterns has the potential to be useful for other rare diseases, especially those that are not measured by registries such as SEER.

Our prospective analysis of clinical trial data identified a higher level of hyperammonemia in FLC patients than currently recognized in clinical practice, suggesting hyperammonemia is an underappreciated source of comorbidity in these patients. We further demonstrate a computational method to assess the mechanism of this paraneoplastic complication, using unsupervised clustering to highlight the similarities between patients with urea cycle disorders and hyperammonemia due to FLC, even with only mild ammonia elevations. Our findings demonstrate the utility of analyzing biochemical phenotypes directly from patient laboratory results. This workflow can serve as a model for developing biochemical insights into disease biology from patient laboratory data and is applicable to rare cancers and other diseases.

There are some limitations in this approach, most importantly the single institution focus of the study. Our adjustments for referral distance are sensitive to the geography of the UCSF catchment area and may not be effectively applied in more densely populated areas. This analysis was further limited by incomplete zip code data. The interplay between the selection bias at a tertiary center and the potential underdiagnosis or failure to diagnose FLC at less expert centers remains unknown.

A comprehensive understanding of rare diseases requires accurate identification of patients—a challenge often compounded by a lack of disease-specific ICD coding. This problem can be self-perpetuating, as imprecise billing data can lead to underestimation of incidence, which decreases prioritization of disease-specific ICD-code creation. Our findings suggest significantly higher national FLC incidence than previous estimates. Furthermore, our approach can capture incidence, detailed clinical information, and potential systemic issues regarding misdiagnosis or underdiagnosis. Integrated analysis of rich computational data sources thus complements patient-led research findings to advance care for patients with rare diseases.

## Methods

### Identification of FLC patients at UCSF

To comprehensively identify and assess the prevalence of patients with FLC within the UCSF medical record, we analyzed four sources of information within the medical records:

1. EPIC hyperspace diagnosis/billing codes (referred to subsequently as EPIC codes)
2. ICD billing codes
3. Histopathology reports on liver biopsy
4. “History and Physical” and Progress notes from physicians in medical subspecialties related to oncological care
5. “History and Physical” and Progress notes from physicians in surgical subspecialties related to hepatobiliary surgery (or inpatient services)

De-identified records for UCSF patients from 2011–2021 were analyzed using Information Commons and under the University of California Institutional Review Board (IRB 19-27988). Due to the de-identified nature of data acquisition and analysis, the requirement for patient consent was waived by the IRB.

For patients with ICD-10 codes for liver cancer between 2012 and 2021, we identified 102 ICD codes for liver conditions that predispose to HCC (Table S1), as well as four ICD codes for non-HCC liver cancers. To increase sensitivity and specificity for FLC diagnoses within our database, we used clinical notes corresponding to patients with liver cancer. We utilized the UCSF Information Commons de-identified notes database, a source of 105 million clinical notes across 2.4 million patients, with patient health information removed to serve as a research tool for clinical hypotheses. To maximize sensitivity, we examined all notes associated with the 9511 patients with an ICD-10 code for liver cancer (“C22.0” and “C22.7”), and we identified all notes for these patients written by a practitioner within a medical or surgical subspecialty that contained the terms “fibrolamellar” or “FLHCC”, (“FLC” was included in searching of histopathology reports). Each patient with at least one note containing one of these terms was identified, and two documents were exported for independent review: (1) the first H&P written for that patient in these departments and (2) the first progress note to contain one of the terms listed above. The goal of this procedure was to identify all patients who *potentially* have diagnoses of FLC within the UCSF EMR, with the accuracy of the diagnosis to be confirmed by manual case review.

The procedure captured 61 patients with a potential diagnosis of FLC through ~50,000 physician notes, 12 patients with potential FLC on the histopathological report, and 30 patients with a CMS code diagnosis of FLC, totaling 69 unique patients. For each of these patients, three independent reviewers reviewed all identified histopathology reports, as well as medical oncology, and surgical notes from patients that contained a mention of FLC, to determine whether the diagnosis of FLC was confirmed within the medical documentation. Of the 69 patients originally identified, a total of 33 were confirmed as having FLC through this approach.

### Estimation of national incidence of FLC

An age-stratified patient cohort was constructed in Komodo’s Healthcare Map^TM^^16^ using the ICD-10-CM inclusion/exclusion criteria verified in the UCSF dataset. We used a Bayesian inference strategy to estimate national incidence, which takes into account the uncertainty inherent in a limited sample size. Using the UCSF comorbidity-excluded HCC patient population defined above, we created a prior distribution for FLC/HCC proportion for each age group between 0–50, in bins of 10 years, using an assumption of the binomial generative model. This maintains the uncertainty of the true FLC proportion of each age group given the limited size of the UCSF dataset, with age groups containing less data having inherently more uncertainty. We then used a Monte Carlo^17^ simulation to generate 10,000 independent populations, with the number of Liver cancer cases within each age range determined by the mean and standard deviation of cases within this age range in the large Komodo comorbidity-excluded dataset between 2017–2019 inclusive. For this purpose, incidence rates for 2020 were excluded due to concern for confounding related to the COVID-19 pandemic. The sum of simulated estimates of FLC patients across age ranges represents the overall incidence expected in the Komodo data. Based on the aggregate payer enrollment captured by the Komodo Healthcare Map^TM^, we extrapolated the expected value and variance of this distribution to estimate national incidence.

### Integration of Zip code data in incidence calculation

In our de-identified patient database, we had access to ZIP3 data (the first three digits of the patient’s address zip code) on the patient’s home address. Unfortunately, we were only about to recover this information for 89% of our patients. None of the patients lost carried a diagnosis of FLC, and we note that, left unaddressed, this would have artificially inflated downstream analyses (see the renormalizing procedure below). To estimate a patient’s home location and distance traveled for care, we took the centroid of the closest five-digit zip code starting with the ZIP3 of that patient and calculated the distance *d* from there to the zip code of the UCSF medical center. Starting from x = 2000 miles from UCSF’s medical center, we reran the simulation described above, only including patients with a distance *d* less than x. To account for the 11% of HCC patients for we were unable to recover Zip code data from, we calculated the fraction of HCC patients still remaining after distance exclusion, and then added that proportion of the patients lost back into the HCC count for that distance, weighted by the age distribution.

### Analysis of hyperammonemia in FLC patients on clinical trial

Baseline and on-treatment ammonia levels were collected for FLC patients participating in the NCT01642186 (start date: July 12, 2012) and NCT02234986 (start date October 2015) trials^18,19^ and are reported here. Written informed consent was obtained for all patients in the two trials above.

### Identification of hyperammonemia patients and clinical labs at UCSF

We identified all clinical encounters where ammonia levels were drawn for patients at UCSF between 2011 and 2021, allowing for multiple encounters for the same patient. For each of these encounters, we identified the maximum ammonia value during that encounter. Using the date and time of collection for this ammonia level, we identified the most temporally proximate blood draw for each lab in a comprehensive metabolic panel (sodium, potassium, chloride, bicarbonate, blood urea nitrogen, creatinine, alanine transaminase, aspartate transaminase, total bilirubin, alkaline phosphatase) and collected their values. The vast majority of encounters had all of these values within 24 h of the ammonia level, but we accepted any encounter that had all of these labs within seven days of the maximum ammonia level. If an encounter did not contain all of these laboratory values within this seven-day window, it was excluded from the analysis.

### Application of machine learning clustering and enrichment

K-means clustering and plots were created using the Python package Scanpy. Using the 3066 encounters with hyperammonemia (ammonia level ≥50) and a complete metabolic panel (as described above). We used the scanpy.pp.scale function to “*z*-score” and normalize these lab values to zero mean and unit variance. We computed the neighborhood graph using a Minkowski distance metric (n_neighbors = 5, n_pcs = 40). To identify related communities in a large graph, we applied Leiden clustering was used with resolution 0.2, leading to eight clusters on these 11 variables^20^. Enrichment of diagnoses within specific clusters were determined by Fisher’s exact test^21^, with a false discovery rate (FDR) estimated by multiplying by # of clusters for single cluster enrichment analysis and by (# of clusters) choosing 2 (= 16) for enrichment analysis of two clusters.

## Supplementary information

Supplementary Information

supplementary table 1

Supplementary table 2

## Data Availability

Due to the rarity of the disease and the limited sample sizes of FLC patients within this cohort, it was deemed unethical to disclose demographic information about patients for fear of re-identification. Data on ICD-code cooccurrence and Hyperammonemia clustering are available from openICPSR via restricted access download at 10.3886/E185781V1. Interested researchers will need to apply to ICPSR with a description of their research, a confidential data security plan, a signed restricted data use agreement, and IRB approval or exemption.

## Code availability

Code for hyperammonemia analysis and incidence estimates available at: https://github.com/orpheus1234/FLC_hyperammonemia.
