# Supplementary material for: Defining incidence and complications of fibrolamellar liver cancer through tiered computational analysis of clinical data
Source: NPJ Precis Oncol. 2023 Mar 23;7:29. doi: 10.1038/s41698-023-00371-2 (PMC10034241; doi:10.1038/s41698-023-00371-2)
Supplement: Supplementary file 1 — Supplementary Information [file 41698_2023_371_MOESM1_ESM.pdf]

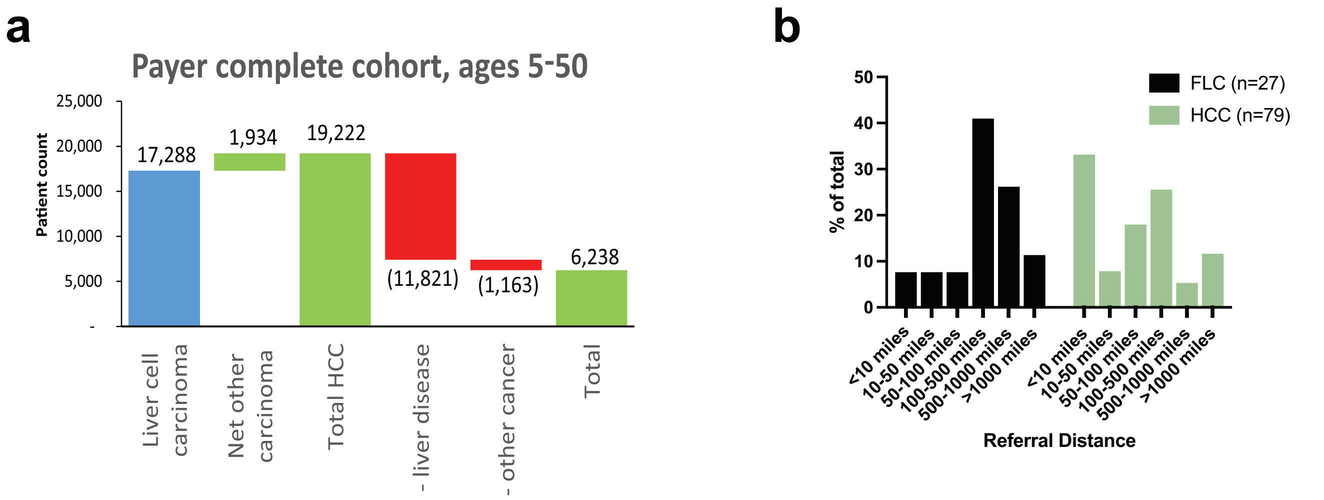

Supplementary Figure 1. Supporting Data for inference of FLC incidence: A. Step-by-step breakdown of the identification of the initial Liver Cancer cohort in the Komodo Health Care Map, showing cohort scale and changes. **B.** Distance from home zip code to UCSF for FLC patients and patients in HCC comparison cohort derived from ZIP3 data.

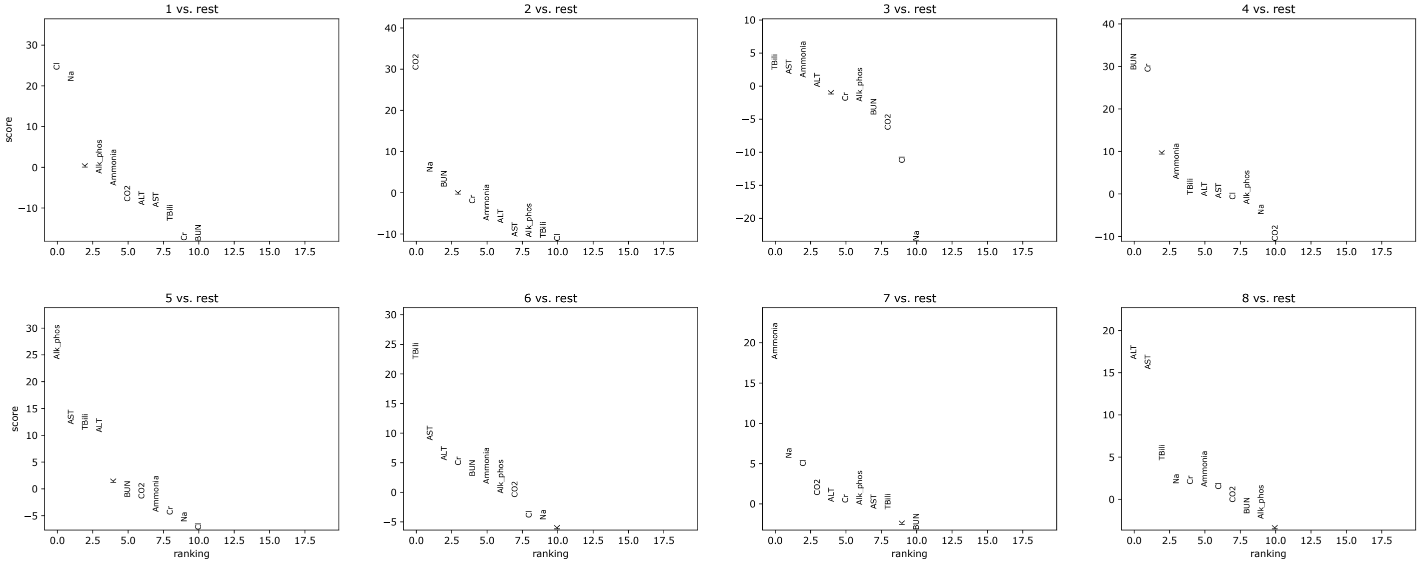

Supplementary Figure 2. Lab-value enrichment within hyperammonemic patient clusters. Lab value differences between clusters were evaluated in a 1 vs. rest Wilcoxon-rank-sum test using the scanpy function rank_genes_test. Y-axis indicates log10(p-value) of difference between test group and rest, with higher absolute values representing significance, positive values indicating higher values in test group and negative numbers indicated lower values in test group
